# Targeting LIM kinases in taxane resistant tumors

**DOI:** 10.18632/oncotarget.10816

**Published:** 2016-07-24

**Authors:** Chloé Prunier, Reuben Kapur, Laurence Lafanechère

**Affiliations:** Team Regulation & Pharmacology of the Cytoskeleton, Institut of Advanced Biosciences, Research Center UGA/INSERM 1209/UMR CNRS, Grenoble, France

**Keywords:** microtubule regulation, breast cancer chemotherapy, LIM kinase

Microtubule stabilizing drugs such as taxanes have proven fairly effective for treating solid tumors, but the mechanism by which these drugs treat cancer is still unsolved. Intratumor imaging, showing that the cell proliferation rate is low in many chemosensitive human cancers, has challenged the dogma that the cytotoxicity of such compounds is the result of their effect on rapidly dividing cells [1]. Moreover, attempts to develop selective antimitotic cancer drugs that inhibit proteins mainly involved in mitosis have failed. These findings suggest that taxanes may target other microtubule-based mechanisms in addition to mitosis, in cancer cells as well as in cells of the tumor environment, or that their therapeutic effect results from a microtubule independent targeting [1, 2]. In addition, taxanes cause severe side effects, including myelosuppression and neurotoxicity, presumably due to the general perturbation of microtubule functions in normal cells. Finally, the fact that many cancers are inherently resistant to taxane chemotherapy or become so during prolonged treatment make these drugs less than ideal.

The search for next generation microtubule stabilizing drugs with increased efficacy is thus intense. Several strategies have been proposed for the development of potentially more effective and less toxic anticancer drugs. One approach is to find drugs that target nonessential proteins, such as microtubule regulators. If such a target would be more active in cancer cells, or centrally involved in cancer aggressiveness, its inhibition would improve drug selectivity for tumors over normal tissue and contribute to a greater therapeutic window.

LIM Kinases (LIMKs) are enzymes whose activity is elevated in cancers compared to normal tissue. LIMKs regulate the architecture of the actin cytoskeleton by phosphorylation and inactivation of actin depolymerization factors of the ADF/cofilin family. Independently of this effect on actin microfilament dynamics, LIMKs also regulate microtubule dynamics, but whether this regulation occurs through a direct binding of LIMK to microtubules or through phosphorylation of an associated protein is still unknown [3, 4]. When LIMKs are inhibited, microtubules are stabilized and actin microfilaments are severed. Owing to their stabilizing effect on microtubules, LIMK inhibitors may provide a therapeutic strategy to treat taxane-resistant cancers. A highly selective LIMK inhibitor, Pyr1, has been previously characterized by Prudent et al [4]. Although ATP-competitive, Pyr1 inhibits only LIMKs out of 110 kinases tested. When applied on cells, Pyr1 stabilizes microtubules, induces a moderate cell cycle arrest at the S-G2/M phase and blocks actin microfilament dynamics and cell motility.

Anticancer drugs are currently classified in two main categories: targeted drugs and cytotoxic drugs. By perturbing a cancer specific pathway that comes down to LIMKs, Pyr1 behaves as a targeted drug. However, because of the induction of cell-cycle arrest and apoptosis, Pyr1 also behaves as a cytotoxic drug, making the mechanism of action of this drug very novel. These properties prompted the investigation of its effect on breast cancer development and the test of the hypothesis that LIMK inhibition could be efficient in paclitaxel resistant cancers. To that aim, Prunier et al. investigated the effect of Pyr1 on the *in vivo* growth of different breast cancer derived cell lines in tumor xenograft models [5]. Pyr1's effect on taxane sensitive cells was found as efficient as paclitaxel to reduce tumor size. Pyr1 was also able to reduce the size of paclitaxel resistant tumors. Contrary to paclitaxel, Pyr1 did not induce detectable adverse side effects. The subsequent analysis of the tumors showed that Pyr1 treatment induced an increase of detyrosinated and acetylated tubulin in tumors, indicative of microtubule stabilization. Thus, one common denominator of the *in vivo* efficiency of paclitaxel and Pyr1 is microtubule stabilization, reinforcing the notion that targeting microtubule dynamics is central to the therapeutic effects of these drugs.

Using fluorescent and luminescent cancer cells, Prunier et al. found that Pyr1 administration has a strong effect on the metastatic load, preventing the growth of metastasis but not their spread. The effect on metastatic load could involve the same mechanisms as those observed in the primary tumor, including an inhibition of integrin-mediated adhesion to the extracellular matrix through an Integrin-Linked-Kinase/β-parvin/LIMK/ cofilin pathway that has been shown to play a central role in tumor initiation and metastatic colonization [6]. Intravital imaging showed that *in vivo* LIMK inhibition has heterogeneous effect on tumor cell motility and did not prevent metastasis spread. This was unexpected as LIMK inhibition is able, *in vitro* to impede cell motility and as it has recently been shown that LIMK is required for matrix degradation through phosphorylation of membrane type 1-matrix metalloproteinase [7]. The complexity of the tumor environment could account for this discrepancy, as well as uncontrolled variations of Pyr1 concentration in animal tissues. Thus, if LIMKs are going to be targeted as anti-cancer agents either as a monotherapy or in combination with existing drugs, a better characterization of their mechanism of action on microtubules and of their different substrates is essential.
